# Supplementary material for: Prevention of Cardiovascular Disease and Cancer Mortality by Achieving Healthy Dietary Goals for the Swedish Population: A Macro-Simulation Modelling Study
Source: Int J Environ Res Public Health. 2019 Mar 12;16(5):890. doi: 10.3390/ijerph16050890 (PMC6427376; doi:10.3390/ijerph16050890)
Supplement: Supplementary file 1 [file ijerph-16-00890-s001.pdf]

International Journal of
Environmental Research and Public Health

MDPI

Supplementary materials 1

Table S1: Age-group and sex-specific mean (standard deviation) dietary intake incorporated in the PRIME model*

Dietary components	Men (n=792)				Women (n=1005)			
Age-group and sample size	18-30 (132)	31-44 (183)	45-64 (308)	65-80 (169)	18-30 (202)	31-44 (247)	45-64 (358)	65-80 (198)
Energy (kcal/day)	2246 (830)	2343 (573)	2254 (674)	2083 (550)	1819 (538)	1820 (517)	1755 (510)	1703 (432)
Vegetables (gm/day)	142 (98)	174 (89)	176 (114)	169 (100)	162 (96)	189 (95)	192 (104)	178 (90)
Fruits (gm/day)	67 (88)	73 (87)	117 (124)	149 (110)	112 (95)	135 (104)	155 (111)	182 (107)
Fibre (gm/day)	18.6 (8.5)	21.3 (7.9)	21.8 (8.0)	22.5 (7.6)	17.3 (6.5)	18.5 (7.3)	19.3 (7.0)	20.0 (6.5)
Salt (gm/day)	9.12 (3.47)	9.55 (2.56)	9.09 (2.91)	8.03 (2.16)	6.92 (2.06)	7.19 (2.18)	6.89 (1.93)	6.36 (1.85)
Fat (%E)	33.9 (6.7)	34.6 (6.4)	33.8 (6.5)	33.6 (6.3)	34.6 (5.7)	34.7 (5.6)	34.7 (7.3)	33.2 (5.5)
Saturated fat (%E)	13.2 (3.4)	13.2 (3.2)	13.0 (3.2)	12.8 (3.2)	13.1 (2.8)	13.3 (3.1)	13.2 (3.7)	12.8 (2.9)
MUFA (%E)	12.8 (2.8)	13.1 (2.8)	12.7 (2.8)	12.5 (2.5)	13.1 (2.7)	13.0 (2.5)	12.9 (3.1)	12.3 (2.5)
PUFA (%E)	5.2 (1.9)	5.6 (2.0)	5.4 (1.8)	5.6 (2.2)	5.8 (2.1)	5.6 (1.9)	5.8 (2.0)	5.5 (1.7)
Cholesterol (mg/day)	314 (172)	319 (139)	322 (141)	321 (138)	245 (121)	257 (113)	274 (129)	269 (123)

*Obtained from "Riksmaten-vuxna 2010-11"

Abbreviations: %E, percentage of total energy; MUFA, Monounsaturated fatty acids; PUFA, Polyunsaturated fatty acids
